# Supplementary material for: Identification of photoperiod-induced specific miRNAs in the adrenal glands of Sunite sheep (Ovis aries)
Source: Front Vet Sci. 2022 Jul 22;9:888207. doi: 10.3389/fvets.2022.888207 (PMC9354845; doi:10.3389/fvets.2022.888207)
Supplement: Supplementary file 1 [file Data_Sheet_1.ZIP › Supplementary file/miRDeep2 file.zip!/miRDeep2 file/Novel_Structure/SPLP42A2/Novel_283.pdf]

Provisional ID	: NC_019469.2_24295
Score total	: 2.1
Score for star read(s)	: -1.3
Score for read counts	: 0
Score for mfe	: 1.8
Score for randfold	: 1.6
Score for cons. seed	:
Total read count	: 39
Mature read count	: 39
Loop read count	: 0
Star read count	: 0

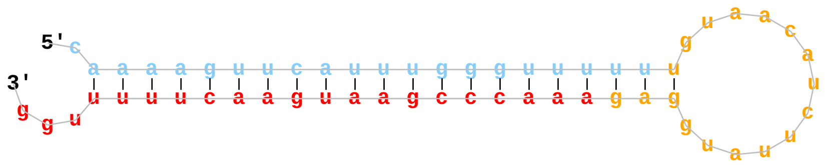

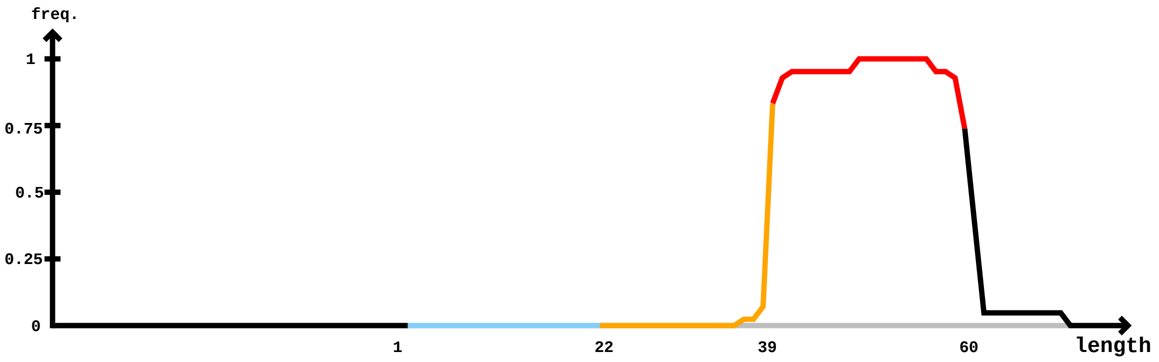

Star Mature

```
5'- uagacggcaugacccuuuuaagguauggguugacccaaaaguucauuuggguuuuuuguaacaucuuauggagaaacccgaaugaacuuuuuggcccacccaguacuaga   -3'    exp
    ((((.((......)).)))).((((((((.(.((.((((((((((((((((((((.............))))))))))))))))))))).)).).))))).))))...    reads      mm         sample
    ......................................................................gagaaacccgaaugaacuuu....................    1          0          seq
    ........................................................................gaaaccUgaaugaacuuuuug.................    2          1          seq
    .........................................................................aaacccgaaugaacuuu....................    1          0          seq
    .........................................................................aaacccgaaugaacuuuuuC.................    1          1          seq
    .........................................................................aaaccAgaaugaacuuuuug.................    1          1          seq
    .........................................................................aaacccgaaugaaAuuuuug.................    1          1          seq
    .........................................................................aaacccgaaugaacuuuuugg................    28         0          seq
    ..........................................................................aacccgaaugaacuuuuu..................    1          0          seq
    ..........................................................................aacccgaaugaacuuuuug.................    2          0          seq
    ..........................................................................aacccgaaugaacuuuuuC.................    1          1          seq
    ...........................................................................acccgaaugaacuuuuuggc...............    1          0          seq
    ...................................................................................ugaacuuuuuggccAacccagu.....    2          1          seq
```
